# Apolipoprotein E-containing HDL decreases caspase-dependent apoptosis of memory regulatory T lymphocytes

**DOI:** 10.1016/j.jlr.2023.100425

**Published:** 2023-08-12

**Authors:** Laura Atehortua, Jamie Morris, Scott E. Street, Nicholas Bedel, W. Sean Davidson, Claire A. Chougnet

**Affiliations:** 1 Division of Immunobiology, Cincinnati Children’s Hospital Research Foundation, University of Cincinnati College of Medicine, Cincinnati, OH, USA; 2 Division of Experimental Pathology, Department of Pathology and Laboratory Medicine, University of Cincinnati, Cincinnati, OH, USA

**Keywords:** HDL, APOE, memory Treg, apolipoproteins, apoptosis, CVD, lymphocytes, atherosclerosis

## Abstract

Plasma levels of HDL cholesterol are inversely associated with CVD progression. It is becoming increasingly clear that HDL plays important roles in immunity that go beyond its traditionally understood roles in lipid transport. We previously reported that HDL interaction with regulatory T cells (Treg) protected them from apoptosis, which could be a mechanism underlying the broad anti-inflammatory effect of HDL. Herein, we extend our work to show that HDL interacts mainly with memory Treg, particularly with the highly suppressive effector memory Treg, by limiting caspase-dependent apoptosis in an Akt-dependent manner. Reconstitution experiments identified the protein component of HDL as the primary driver of the effect, though the most abundant HDL protein, apolipoprotein A-I (APOA1), was inactive. In contrast, APOE-depleted HDL failed to rescue effector memory Treg, suggesting the critical role of APOE proteins. HDL particles reconstituted with APOE, and synthetic phospholipids blunted Treg apoptosis at physiological concentrations. The APOE3 and APOE4 isoforms were the most efficient. Similar results were obtained when lipid-free recombinant APOEs were tested. Binding experiments showed that lipid-free APOE3 bound to memory Treg but not to naive Treg. Overall, our results show that APOE interaction with Treg results in blunted caspase-dependent apoptosis and increased survival. As dysregulation of HDL-APOE levels has been reported in CVD and obesity, our data bring new insight on how this defect may contribute to these diseases.

## Graphical abstract

**Figure:**
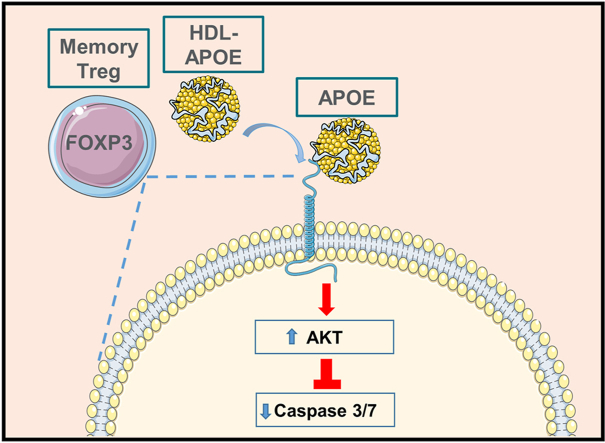

Plasma levels of HDLs are associated with protection from CVD, inflammatory and metabolic diseases such as obesity (1, 2), but the underlying mechanisms of how HDL exerts their immunoprotective effects are not fully understood. Traditionally, HDL was thought to mainly carry lipids in reverse cholesterol transport (3), but only about one-third of the 250 or so proteins that associate with HDL play known roles in lipid transport. In contrast, the majority play roles in modulating inflammation and the innate immune system, strongly implying important roles in host defense (4). HDL is known to display important immunomodulating functions by interacting with many immune cells, but most of the work has focused on myeloid cells (5). We recently found that HDL could directly interact with human regulatory T cells (Treg) and promote their accrual (6). Treg also limit vascular endothelial injury by decreasing the accumulation of proinflammatory cells and by producing anti-inflammatory cytokines in the atherosclerotic plaque (7). Given the known Treg atheroprotective effect, this newly discovered function of HDL could also play an important role. However, the amplitude of HDL antiapoptotic effect for Treg varied widely between individuals (6).

Human Treg are phenotypically and functionally heterogeneous (8, 9, 10, 11), with different subsets with unique specificities and immunomodulatory functions (8, 9). Three major subsets can be identified in the peripheral blood: naïve Treg (nTreg), memory Treg (mTreg), and effector memory Treg (emTreg). Treg, notably the highly functional emTreg subset, are essential for maintaining immune homeostasis, protection against CVD, and complications related to metabolic disorders (12). Thus, we posited that these two separate anti-inflammatory processes, HDL immunomodulatory properties, and Treg anti-inflammatory function, may work in concert to limit CVD and metabolic disorders.

HDLs are heterogenous particles, consisting of a central core of neutral lipid esters, surrounded by a monolayer of phospholipids, free cholesterol, and apolipoprotein functions (3, 13). Both HDL lipids and apolipoproteins have been shown to be involved in the HDL antiapoptotic effect for endothelial cells (14, 15). Between the apolipoproteins, APOA1 is the most abundant protein, and lipid-free APOA1 favored Treg expansion in a murine model of autoimmunity (16). APOA1 could also inhibit the differentiation of Treg into atherogenic T cells (17). Besides APOA1, APOE, one of the minor proteins of the HDL, has been significantly associated with lower risk of coronary heart disease, and it also promotes metabolic steps in reverse cholesterol transport (18). Interestingly, Apoe(−/−) mice exhibit significantly decreased Treg numbers (19, 20, 21) and secretion of Treg-related cytokines, notably of transforming growth factor-beta (22).

Herein, we found that HDLs preferentially bind to mTreg and emTreg and promote their survival by decreasing caspase-dependent apoptosis in an Akt-dependent manner. Our results also show that HDL apolipoproteins, particularly APOE, drive emTreg and mTreg survival. This mechanism could thus contribute to the atheroprotective and anti-inflammatory properties of HDL, through their effect on Treg homeostasis.

## Materials and methods

### Cell isolation

Peripheral blood mononuclear cells from healthy donors (Hoxworth blood center, Cincinnati, OH) were separated by centrifugation through Ficoll-Hypaque (GE, Fairfield, CT). CD4+ T cells were purified by negative selection using the Miltenyi CD4 negative separation kit (Auburn, CA), per the manufacturer’s instructions. Aliquots of purified CD4+ T cells were frozen in FBS + 10% DMSO and stored in liquid nitrogen. The viability of thawed cryopreserved cells was >90%. To isolate bulk Treg, cryopreserved CD4+ T cells were stained with live dead aqua (ThermoFisher, Waltham, MA), anti-CD8-FITC, anti-CD25-APC (BD Pharmingen, San Diego, CA), anti-CD127-PE (Beckman Coulter, Fullerton, CA), and sorted using a FACS Aria (BD) (supplemental Fig. S1A). Purity of the sorted Treg was >90%, as determined by postsorting analysis of FOXP3 expression (supplemental Fig. S1B). FOXP3 expression was also significantly higher in Treg compared with conventional CD4+T cells (non-Treg) after sorting (supplemental Fig. S1C). In some experiments, Treg subsets (nTreg, mTreg, and emTreg) were isolated, briefly cryopreserved CD4+ T cells were stained with anti-CD8-FITC, anti-CD25-APC, anti-HLA-DR-APC-Cy7 (BD), anti-CD127-PE (Beckman Coulter), anti-CD45RA-PB, anti-CD95-BV510 (BioLegend, San Diego, CA), and sorted using an FACS Aria (supplemental Fig. S1A).

### Reconstitution of HDL lipids and POPC into HDL particles

HDL lipids were isolated from total HDL (Sigma-Aldrich, St. Louis, MO) using a Bligh-Dyer lipid extraction method (23). Polar lipids were isolated using thin layer chromatography, reconstituted in chloroform, and a phosphorous assay determined the HDL lipid concentration. A modified Bio-bead sodium cholate removal method was used for the preparation of reconstituted HDL (rHDL) discs as reported (24). Phospholipid POPC (Avanti Polar Lipids, Birmingham, AL) was used at a molar ratio of 85:1 POPC:APOA1 and 72:13:1 POPC:HDL lipids:APOA1 to generate 96 Å particles containing either HDL lipids or POPC. Particle size and homogeneity was determined by 4–15% nondenaturing gradient gel electrophoresis (Mini-PROTEAN TGX; Bio-Rad) with Coomassie blue staining (supplemental Fig. S2A). Protein concentrations were determined by the modified Markwell-Lowry assay.

### Generation of apo HDL, rHDL-APOA1, rHDL-APOA2, rHDL-APOA4, rHDL-APOE2, rHDL-APOE3, and rHDL-APOE4 discs

HDL proteins from human HDL isolated from fresh plasma were isolated using a Bligh-Dyer lipid extraction method (23). rhAPOA1, rhAPOA2, rhAPOA4, rhAPOE2, rhAPOE3, and rhAPOE4 (recombinant human forms) were expressed in cells, purified by 6x His tag followed by proteolytic removal of the N-terminal tag as described previously (25, 26). Human HDL was delipidated in ethanol/diethyl ether as described by Scanu and Edelstein (27). The reconstitution of HDL and recombinant proteins into HDL particles was performed as described before for the HDL lipids and POPC into HDL particles (24, 28). Briefly, the proteins were reconstituted with POPC and free unesterified cholesterol (Sigma, St. Louis, MO) at molar ratio of 85:1 POPC:APOA and 150:1 POPC:APOE (isoforms stated above) to generate 96 Å particles. The particle size and homogeneity were confirmed by a 8–25% nondenaturing gradient gel electrophoresis with Coomassie blue staining and compared against an SDS 4–15% gel (Mini-PROTEAN TGX; Bio-Rad) with Coomassie blue staining (supplemental Fig. S2B–D). Protein and particle concentrations were determined by a modified Markwell-Lowry assay. Phospholipid concentrations were determined using a modified choline-containing phospholipid colorimetric assay (Fujifilm; Wako).

### Generation of rhAPOA1, rhAPOE2, rhAPOE3, and rhAPOE4

pET-32 APOE3 plasmid was used as a template to construct a mutant plasmid by site-directed mutagenesis to create the plasmid for the recombinant APOE2 isoform. The site-direction mutagenesis was accomplished by changing arginine 158 to a cystine. The pET-30 APOA1 and pET-32 APOE isoforms (APOE2, APOE3, and APOE4) (25) were expressed and purified as described previously (25). Prior to use, the purified APOA1, APOE3, and APOE4 isoforms were resolubilized in 3 M guanidine HCl followed by refolding at 4°C by buffer exchange into PBS. The purified APOE3 was resolubilized in 3 M guanidine HCl followed by a refolding at 4°C by buffer exchange into PBS with 5 mM DTT (ThermoFisher) (supplemental Fig. S2C,D). After purification, rhAPOA1 and rhAPOE3 were labeled at a 1:1 mass:mass ratio using Alexa Flour 488 5-TFP (Invitrogen) stirring at room temperature for 1 h. Labeled protein was purified away from unreacted label using a HiLoad 16/600 Superdex (Cytiva, Washington, DC) equilibrated in PBS. Peak fractions were pooled and concentrated and sterile filtered. Apolipoprotein concentration was determined again by using a modified Markwell-Lowry assay. Fluorescence was confirmed using SDS gel exposed to UV.

### Depletion of APOE from total HDL

APOE-depleted HDL was prepared using immunoaffinity chromatography. Briefly, HDL (Sigma-Aldrich) was applied to an immunoaffinity chromatography column containing affinity-purified anti-APOE antibody (Academy Biomedical, Houston, TX) covalently bound to Sepharose 4B resin on a fast protein liquid chromatography system at 0.5 ml/min in PBS buffer containing 140 mM NaCl, 0.01% EDTA, and 0.01% azide (pH = 7.4). Bound APOE-HDL was eluted in 3 M sodium thiocyanate in PBS and immediately desalted using polyacrylamide 6000 columns (Thermo Scientific). Flow thru (APOE-depleted HDL) was then concentrated and analyzed by Western blot to confirm the depletion of APOE from the original total HDL sample. Briefly, 2 μg of total protein for either APOE-depleted HDL or total HDL was probed using an affinity-purified HRP-conjugated goat anti-APOE primary antibody (Meridian Life Science) at a 1:2,500 dilution.

### Culture conditions

Purified Treg cells were cultured in the serum-free medium, X-VIVO 15, which contains human albumin, human insulin, human transferrin, and l-glutamine (Lonza, Charleston, TN), overnight at 37°C in the presence or the absence of pooled human HDL (700 μg/ml of protein; Sigma-Aldrich) or different HDL preparations (700 μg/ml of protein) as described in the legends. In some experiments, purified Treg cells were pretreated with either MK-2206 (Akt inhibitor, 3 μM) (MCE, Monmouth Junction, NJ) or the receptor-associated protein (RAP, LDL receptor [LDLR]–related protein [LRP] inhibitor, 10 μM) (Innovative Research, Novi, MI) in X-VIVO medium for 1 h at 37°C. HDL (700 μg/ml of protein) was added, and the cells were incubated overnight.

### Flow cytometry and microscopy analyses

Apoptosis was quantified using cellevent caspase 3/7 green detection reagent (ThermoFisher). Cells were incubated with 3 μM of the reagent for 30 min at 37°C. To separate out nTreg, mTreg, and emTreg, cells were blocked with PBS 2% FBS + human IgG 10 μg/ml and stained with the surface antibodies CD45RA-pacific blue, CD95-BV510 (BioLegend), and HLA-DR-APC-Cy7 (BD) for 30 min at room temperature. Then, cells were fixed and permeabilized with FOXP3 perm/fix buffer kit (ThermoFisher) for 1 h at 4°C, followed by intracellular staining with anti-Ki67-PerCP-Cy5.5 (BD), anti-ICOS-BV605, and anti-CTLA4-BV711 (BioLegend). Samples were acquired on a Fortessa flow cytometer (BD). Flow cytometry data were analyzed using the Flowjo software, version 10.6.2 (BD). For some experiments, bulk Treg cells were cultured in X-VIVO media in the presence of caspase 3/7 green reagent and anti-CD45RO (Alexa Fluor 700; BioLegend) and followed for 24 h by time-lapse imaging, using Nikon AXR Inverted Confocal Laser Scanning Microscope (Nikon Corporation, Minato City, Tokyo, Japan).

### Imaging flow cytometry

To measure HDL binding, nTreg, mTreg, and emTreg cells were sorted from total CD4+ T cells and cultured for 1 h at 4°C or 37°C with HDL-Dil or APOA1-Alexa Fluor 488 or APOE3-Alexa Fluor 488 in X-VIVO 15 media. In some experiments, cells were pretreated with RAP (10 μM) in X-VIVO medium for 1 h at 37°C, before adding the labeled APOE3. Then, for surface and nuclear staining, cells were washed and stained with anti-CD4-AF700 (BioLegend) and NucBlue (ThermoFisher) for 30 min at room temperature. Samples were acquired with the Amnis Image Stream, using 60X objective lens, and analyzed using the IDEAS software (Luminex, Austin, Tx).

### Statistics

Graphics and Statistical analyses were generated with GraphPad Prism software, version 9.3.1 (GraphPad Software, Inc). Most analyses, when the effect of HDL or rHDL-apolipoproteins was compared between the different Treg subsets, used a repeated-measures two-way ANOVA with matched values and Sidak’s multiple comparison test with a single pooled variance. One-way ANOVA test and Sidak’s multiple comparison test with a single pooled variance was used to compare the effect of rHDL-E-POPC and rhAPOE in Treg cells. In some cases, paired *t*-tests were utilized to compare the effect between two groups of treatment. In all cases, a threshold of *P* ≤ 0.05 was considered significant.

## Results

### HDL improves mTreg and emTreg survival

**Fig. 1:**
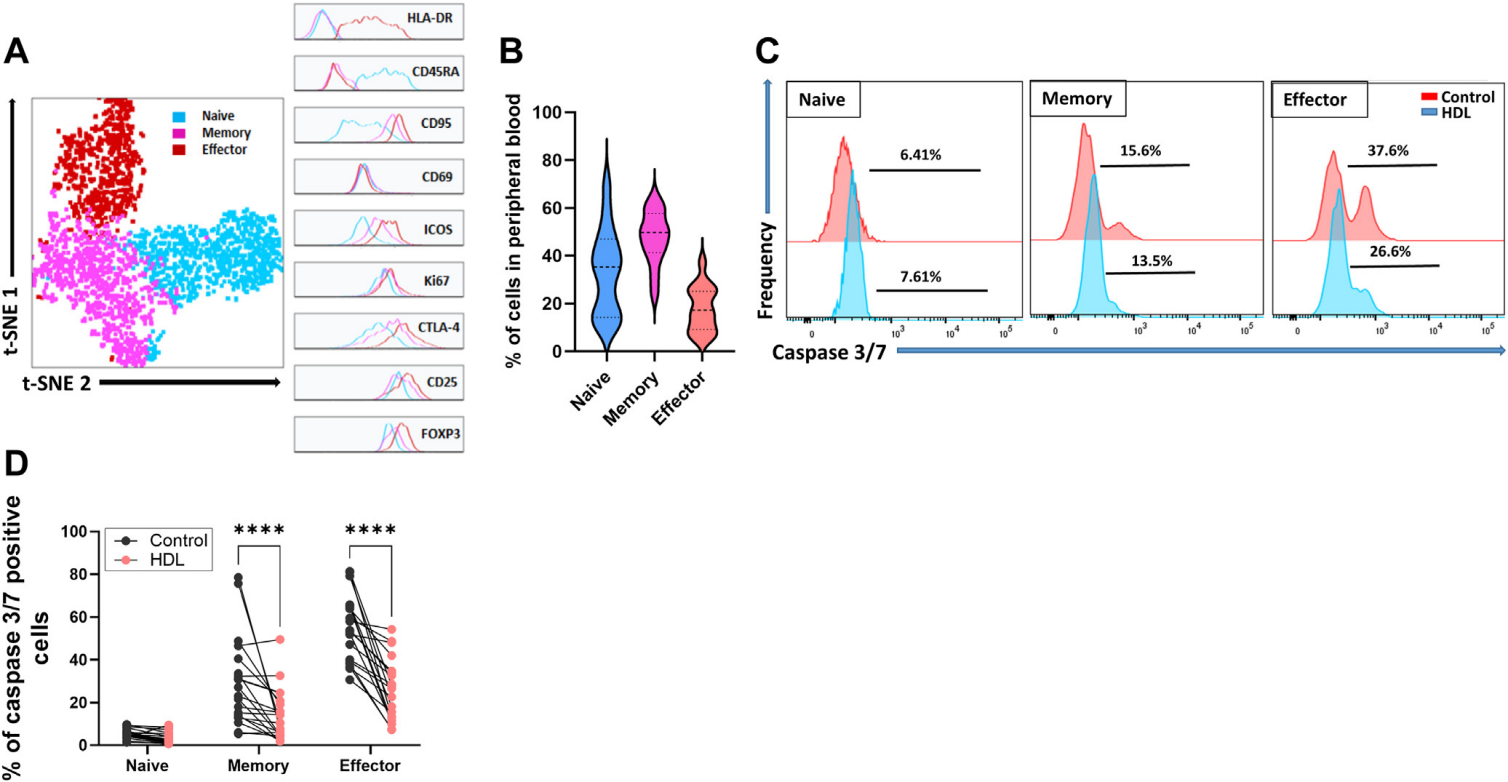
HDL rescues mTreg and emTreg from apoptosis. A: CD4+ T cells were purified by negative bead selection from PBMCs and stained with fluorescently labeled antibody. Within CD4+ T cells, Treg were identified as CD3+CD4+FOXP3+ within the live/dead singlet cells. Dimension reduction analysis was performed by *t*-distributed stochastic neighbor embedding. A representative example of 15 experiments is shown. B: Frequency of nTreg, mTreg, and emTreg in peripheral blood (n = 15). C and D: Bulk Treg were purified by cell sorting from CD4+ T cells from healthy donors and incubated overnight with or without pooled HDL (700 μg/ml of protein) in X-VIVO medium. Caspase-3/7 expression was measured by flow cytometry in nTreg, mTreg, and emTreg. C: Representative example of caspase 3/7 staining in Treg subsets before and after HDL treatment. D: Effect of HDL on caspase 3/7 expression in Treg subsets. Each line represents cells from one individual (n = 20). Asterisks indicate significant differences (∗∗∗∗ *P*< 0.0001) two-way ANOVA.

In our previous work (6), we noted significant intersubject variability in the response of their bulk Tregs to a common HDL sample. We speculated HDL may differentially affect Treg subsets, which can vary in distribution across individuals. We thus characterized Treg subsets and their response to HDL. Three different circulating Treg subsets can clearly be identified via cellular markers, as reported in the literature (8, 9, 10, 11): nTreg, mTreg, and emTreg (Fig. 1A). In addition to differential expression of CD45RA on nTreg, and CD95 on mTreg, only emTreg express high levels of the activation marker HLA-DR. mTreg, particularly emTreg, also express the highest levels of the suppressive molecules, CTLA-4, ICOS, CD25, as well as high Ki67 and FOXP3 levels (Fig. 1A), further suggesting that emTreg are the most activated and suppressive subset. nTreg and mTreg are more abundant in peripheral blood than emTreg (Fig. 1B), suggesting that only a low number of nTreg or mTreg differentiate into emTreg.

To assess whether HDL differentially affect Treg subsets, Treg cells were cultured overnight in the serum- and cytokine-free X-VIVO medium either alone or in the presence of HDL, and the expression of proapoptotic caspase-3/7 was measured after 24 h. This ex vivo assay has been shown to correlate well with T-cell in vivo survival (29). After 24 h, mTreg, particularly the effector memory subset, expressed higher levels of caspase-3/7 than nTreg (Fig. 1C,D). The highest difference in caspase-3/7 expression between nTreg and mTreg was observed after 14 h and sustained up to 24 h (supplemental Fig. S3). Treg subsets maintained their phenotype after 24 h of culture, including in the presence of HDL treatment (supplemental Fig. S4). Consistent with their low basal levels, HDL incubation did not significantly affect nTreg expression of caspase-3/7. However, HDL dramatically reduced caspase-3/7 expression in mTreg and emTreg subsets after 24 h of culture (Fig. 1C,D), suggesting HDL is antiapoptotic for the most activated and suppressive Treg subsets.

### emTregs and mTregs bind HDL more efficiently than nTregs

**Fig. 2:**
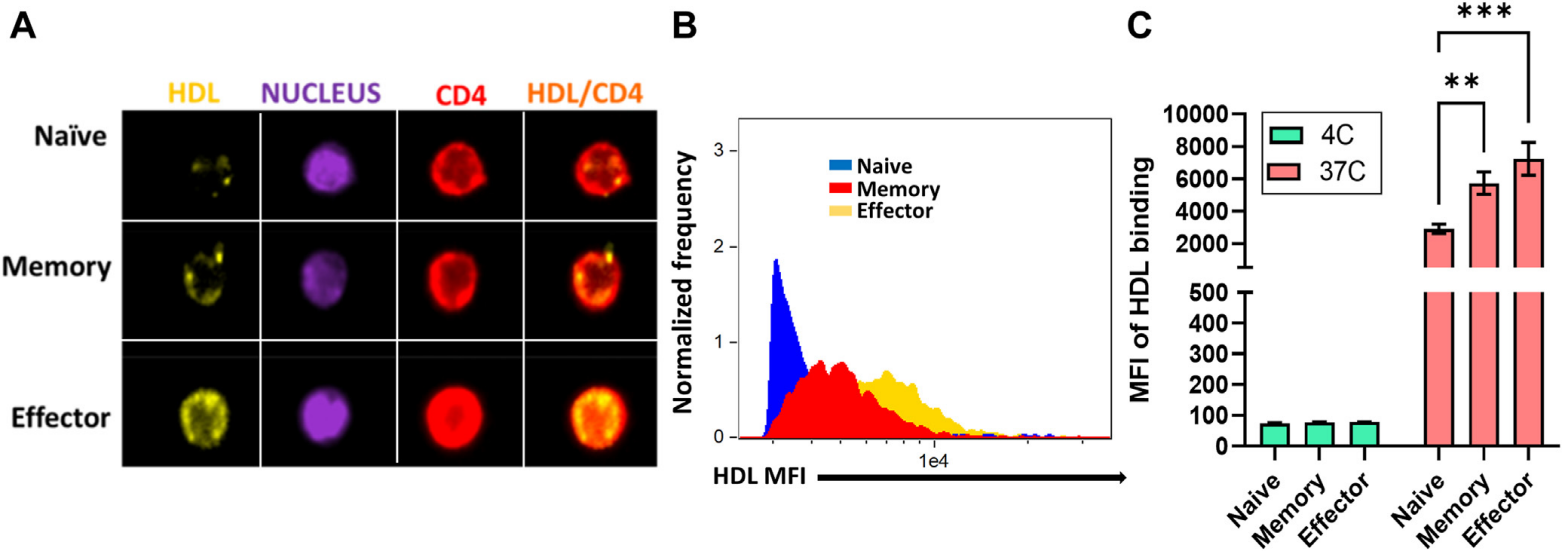
mTreg and emTreg bind higher amounts of HDL than nTreg. Purified Treg subsets from healthy controls were incubated with HDL-DiI in X-VIVO medium at 4 or 37°C. Cells were stained with anti-CD4 and NucBlue to visualize membrane (red) and nucleus (purple) and analyzed by Imagestream. A: Representative example of staining of one cell in each subset. B: MFI of HDL-DiI in all three subsets is shown for one representative individual. C: MFI of HDL-DiI binding in each condition (n = 3). Asterisks indicate significant differences (∗∗*P* < 0.005, ∗∗∗*P* < 0.0005) in two-way ANOVA.

Next, we assessed the ability of different Treg subclasses to bind fluorescently labeled HDL. Figure 2A and supplemental Fig. S5 show that HDL bound minimally to nTregs but exhibited significant binding to mTregs and emTregs at 37°C, with the latter subset exhibiting the most pronounced binding (Fig. 2A–C). Mean fluorescent intensity (MFI) of HDL binding per cell was both high in mTregs and emTregs (Fig. 2B,C). In contrast, HDL bound poorly to all cell types at 4°C (Fig. 2C), indicating that HDL preferentially binds and rescues mTreg and emTreg and does so only at temperatures when typical membrane receptor turnover processes are functional.

### HDL antiapoptotic effect involves the Akt signaling pathway

**Fig. 3:**
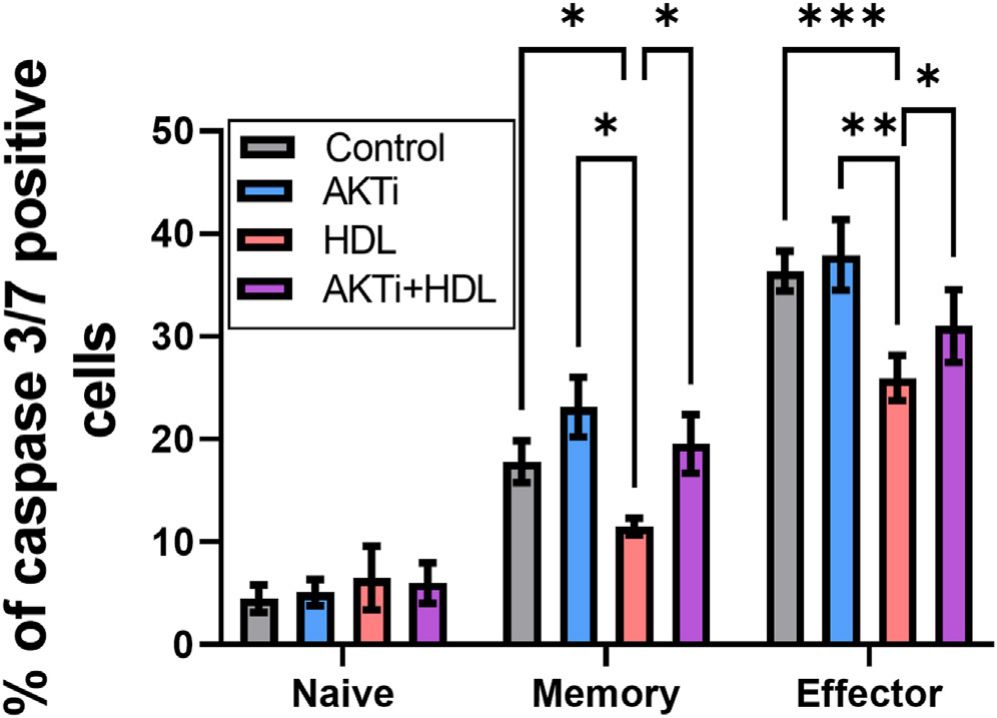
Akt blockage attenuates the HDL antiapoptotic effect on mTreg and emTreg. Purified bulk Treg subsets from healthy controls were pretreated with MK-2206 (Akt inhibitor [AKTi], 3 μM) in X-VIVO medium for 1 h at 37°C. HDL (700 μg/ml of protein) was added, and the cells were incubated overnight. Caspase 3/7 expression of Treg subsets is shown in the figure. Asterisks indicate significant differences (∗*P* < 0.05, ∗∗*P* < 0.005, ∗∗∗∗*P* < 0.0001) in paired *t*-test (n = 7).

The Akt signaling pathway promotes survival of many cell types (30) and has been implicated in previously observed HDL-mediated antiapoptotic effects in endothelial cells (31). Therefore, we investigated its involvement in HDL inhibition of Treg apoptosis. Treg were pretreated for 1 h with the Akt inhibitor, MK-2206 (32), at a dose that did not increase caspase-3/7 activation in Treg subsets compared with the untreated cultures (Fig. 3). Importantly, Akt inhibition significantly decreased HDL antiapoptotic effect in mTreg and emTreg (Fig. 3), suggesting the involvement of this pathway in the HDL protective effect for Treg.

### HDL apolipoproteins, but not those of the A class, rescue mTreg and emTreg from caspase-dependent apoptosis

**Fig. 4:**
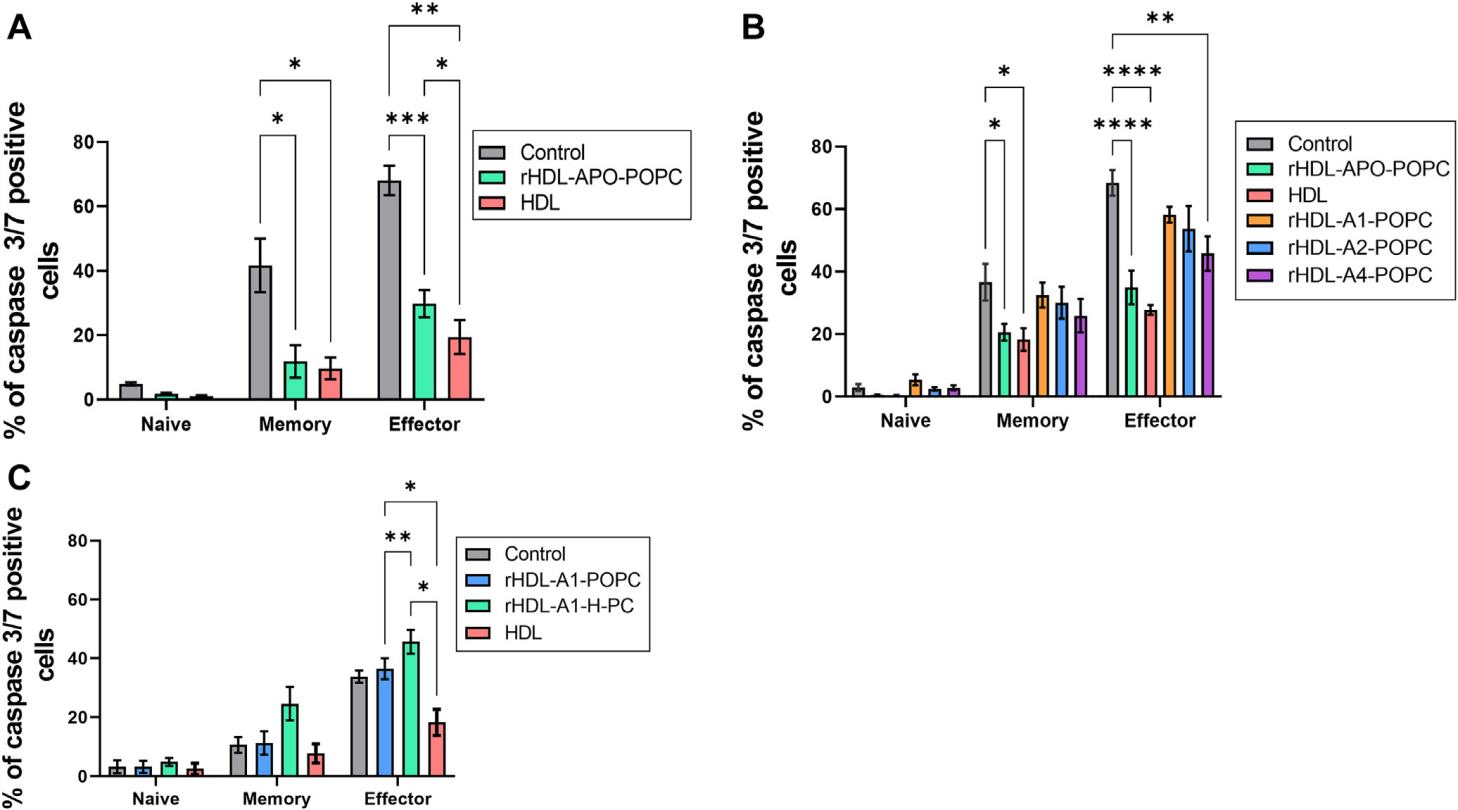
HDL apolipoproteins, but not APOAs, promote survival of mTreg and emTreg from healthy individuals. Caspase 3/7 expression of Treg subsets incubated overnight in X-VIVO medium with (A) PBS (control), apolipoproteins of HDL in POPC (rHDL-APO-POPC, 700 μg/ml of protein) and total HDL (700 μg/ml of protein). Asterisks indicate significant differences (∗∗∗*P* < 0.0005) in two-way ANOVA (n = 6). B: rHDL-APOA(1,2,4)-POPC discs, rHDL-APO-POPC, or total HDL. Asterisks indicate significant differences (∗∗∗∗*P* < 0.0001) in two-way ANOVA test (n = 5). C: APOA1 reconstituted either in POPC (rHDL-A1-POPC) or HDL lipids (rHDL-A1-H-PC). Asterisks indicate significant differences (∗*P* < 0.05 and ∗∗*P* < 0.005) in two-way ANOVA test (n = 3).

HDLs contain more than 250 different proteins and upward of 200 different species of neutral or polar lipids (3, 33). To determine whether the antapoptotic effect of HDL was due to the protein or the lipid component, we took advantage of our ability to selectively incorporate various HDL protein and lipid components into rHDL particles (26, 34). First, we evaluated the protein component of HDL by delipidating HDL under conditions that preserve the protein components (23). We refer to this fraction as apo-HDL because it consists of all the “apo” and other proteins known to be associated with HDL. The apo-HDL fraction was reconstituted with synthetic POPC to form discoidal rHDL particles that are within the size range of native HDL particles (supplemental Fig. S2A). The rHDL particles containing the complement of the native HDL particles, HDL proteins (rHDL-APO-POPC), provided clear protective effects for both mTreg and emTreg subsets (Fig. 4A), suggesting that the protein and not the lipid component mediates HDL-Treg prosurvival effect. To identify the apoprotein(s) involved, we then produced rHDL including isolated apolipoproteins that are known to be relatively abundant in HDL, starting with the APOA class. rHDL particles were produced with APOA1 (the major protein component of HDL), APOA2 the second most abundant apolipoprotein in HDL, and APOA4 another relatively abundant APOA-related protein in HDL. These particles also shared general size and composition with native HDL (supplemental Fig. S2B). As expected, native HDL and rHDL particles containing HDL proteins were effective, whereas neither APOA1 nor APOA2-POPC containing rHDL had a significant protective effect. APOA4 (rHDL-A4-POPC) showed a moderate protective effect for the emTregs (Fig. 4B).

To confirm the pre-eminent role of apolipoproteins, and not HDL lipids, human plasma polar lipids (i.e., phospholipids, sphingomyelins, carotenoids, free cholesterol, etc.) were isolated from neutral lipid esters by thin layer chromatography and combined with APOA1 to produce rHDL particles (rHDL-A1-H-PC). These exhibited size and physical properties similar to native HDL (supplemental Fig. S2A) yet exhibited no ability to protect either mTreg population, similar to particles containing APOA1 and POPC (rHDL-A1-POPC) (Fig. 4C). Together, these data strongly support the conclusion that the lipid component of HDL plays a minimal role in the protective effect for Treg and further implicate the involvement of apolipoproteins other than the major HDL scaffold proteins APOA1 and APOA2.

### APOE-HDLs promote mTreg and emTreg survival

**Fig. 5:**
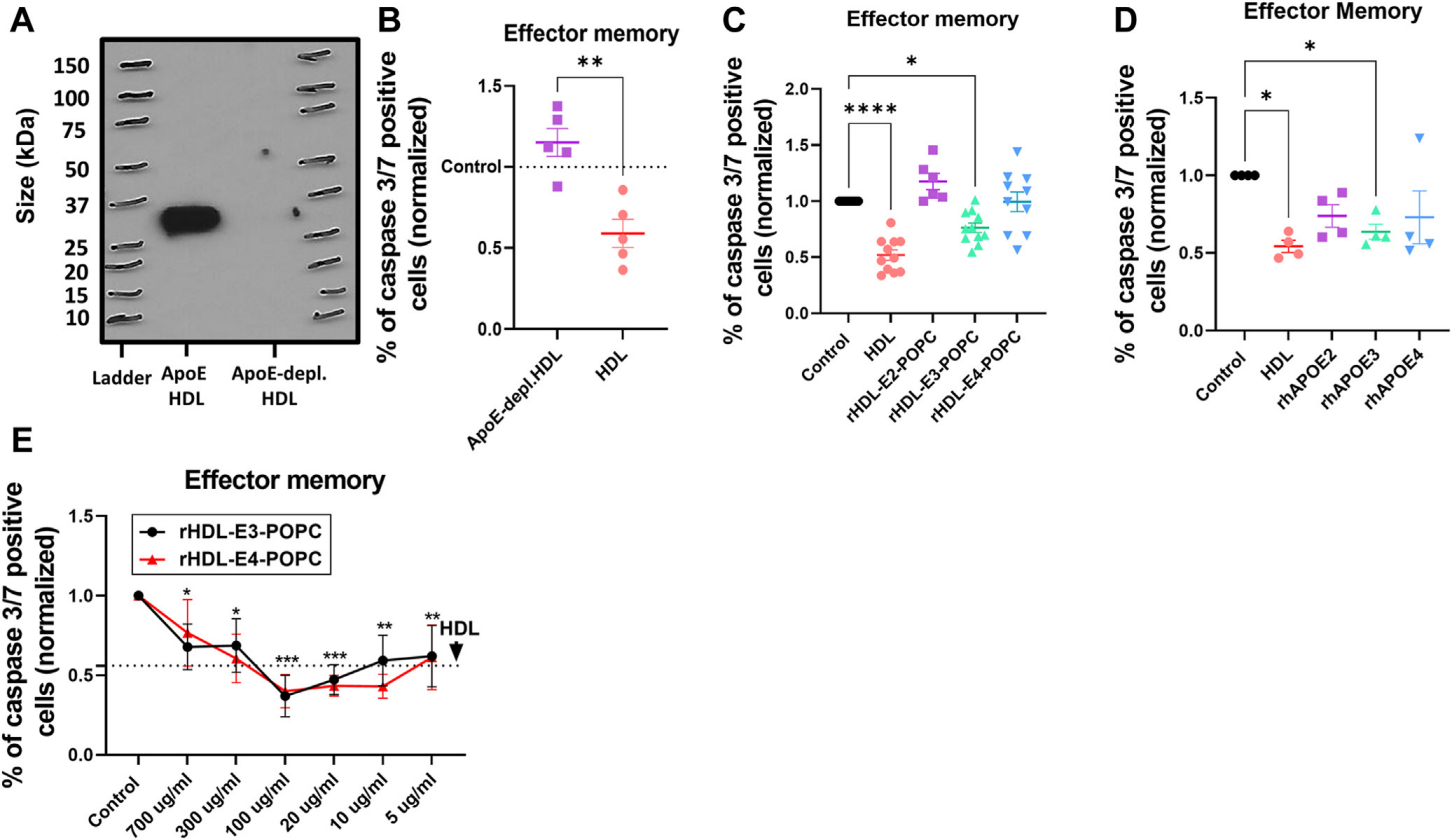
APOE-containing rHDL and lipid-free rhAPOE, but not APOE-depleted HDL, decrease emTreg apoptosis. Purified bulk Treg subsets were incubated with total HDL or APOE-depleted HDL, APOE(2–4)-containing rHDL, or lipid-free rhAPOE(2–4), overnight in X-VIVO medium. A: 4–15% SDS-PAGE analysis of APOE-containing HDL (APOE HDL, lane 1) and APOE-depleted HDL (APOE-depleted HDL, lane 2). B: Caspase 3/7 expression of emTreg incubated with APOE-depleted HDL or total HDL. The data are normalized with respect to the level of caspase expression in the control (PBS). Asterisks indicate significant differences (∗∗*P* < 0.005) in *t*-test (n = 5). Caspase 3/7 expression of emTreg after treatment with (C) rHDL-APOE(2–4)-POPC and (D) rhAPOE(2–4). The data are normalized with respect to the level of caspase expression in the control (PBS). Asterisks indicate significant differences (∗*P* < 0.05, ∗∗∗*P* < 0.001) in one-way ANOVA test (n = 4–6). E: Caspase 3/7 expression of emTreg after the treatment with different concentrations of rHDL-APOE(3,4)-POPC, dotted line shows the mean of total HDL treatment (700 μg/ml of protein). Asterisks indicate significant differences (control vs. rHDL-E-POPC) in two-way ANOVA (∗*P* < 0.05, ∗∗*P* < 0.005, ∗∗∗*P* < 0.0005) (n = 5).

Another relatively abundant HDL constituent is APOE. APOE^−/−^ mice have been reported to have low number of systemic Treg (22, 35) and on plaques (36). To test whether APOE mediated the protective effect seen in the apo-HDL preparations, we used immobilized anti-APOE antibodies to deplete native human HDL particles of those that contain APOE, typically about 10% of native HDL particles (3). SDS-PAGE analysis showed that our depletion protocol removed nearly all Western blot-detectable APOE from total HDL preparations (Fig. 5A). We then compared the effect of total HDL to APOE-depleted HDL on an equal total protein basis (700 μg/ml). Our Treg protection assay shows that APOE-depleted HDL not only failed to protect emTreg from cell death, but it might also be slightly toxic to the cells, whereas the parent native HDL preparation showed the expected protection (Fig. 5B).

In humans, APOE exists in three major isoforms, APOE2, APOE3, and APOE4, which differ by the presence of Cys residues at two sites in the sequence. Taking advantage of our bacterial expression system, we produced APOE2, APOE3, and APOE4-containing rHDL particles with POPC and tested them in our Treg protection assay, at an equalized concentration of 700 μg/ml of total protein. APOE3-containing rHDL exhibited a significant protective effect for the emTreg, whereas APOE4-containing rHDL trended toward a protective effect, but this was not statistically significant. APOE2 in rHDL showed no effect (Fig. 5C). Next, we asked whether APOE (2, 3, 4) proteins could also protect Treg when not complexed to lipids as rHDL particles. rhAPOE3 significantly protected emTreg, similarly to the antiapoptotic effect of the HDL. rhAPOE4 again trended toward a protective effect, as did APOE2, but neither effect was significant because of higher variability (Fig. 5D). APOE3-containing HDL and rhAPOE3 also exhibited an antiapoptotic effect for mTreg (supplemental Fig. S6A,B). In physiological conditions, a --normolipidemic human has about 20–70 μg/ml of circulating APOE (37). Therefore, we performed dose-response experiments with APOE3- and APOE4-containing rHDL to test whether physiological concentrations would protect Treg from apoptosis. Interestingly, lower concentrations of APOE3 and APOE4-containing rHDL showed a significant protective effect on emTreg, including at physiological levels (∼20 μg/ml), an effect similar to that of total HDL (Fig. 5E). Similar results were observed for mTreg, where APOE3 and APOE4-containing rHDL were both more protective at lower concentrations (supplemental Fig. S6C). Taken together, the data strongly implicate APOE, particularly APOE3 and APOE4, as a major operational factor underlying the HDL-mediated mTreg and emTreg survival.

### Lipid-free APOE3 efficiently binds to mTreg

**Fig. 6:**
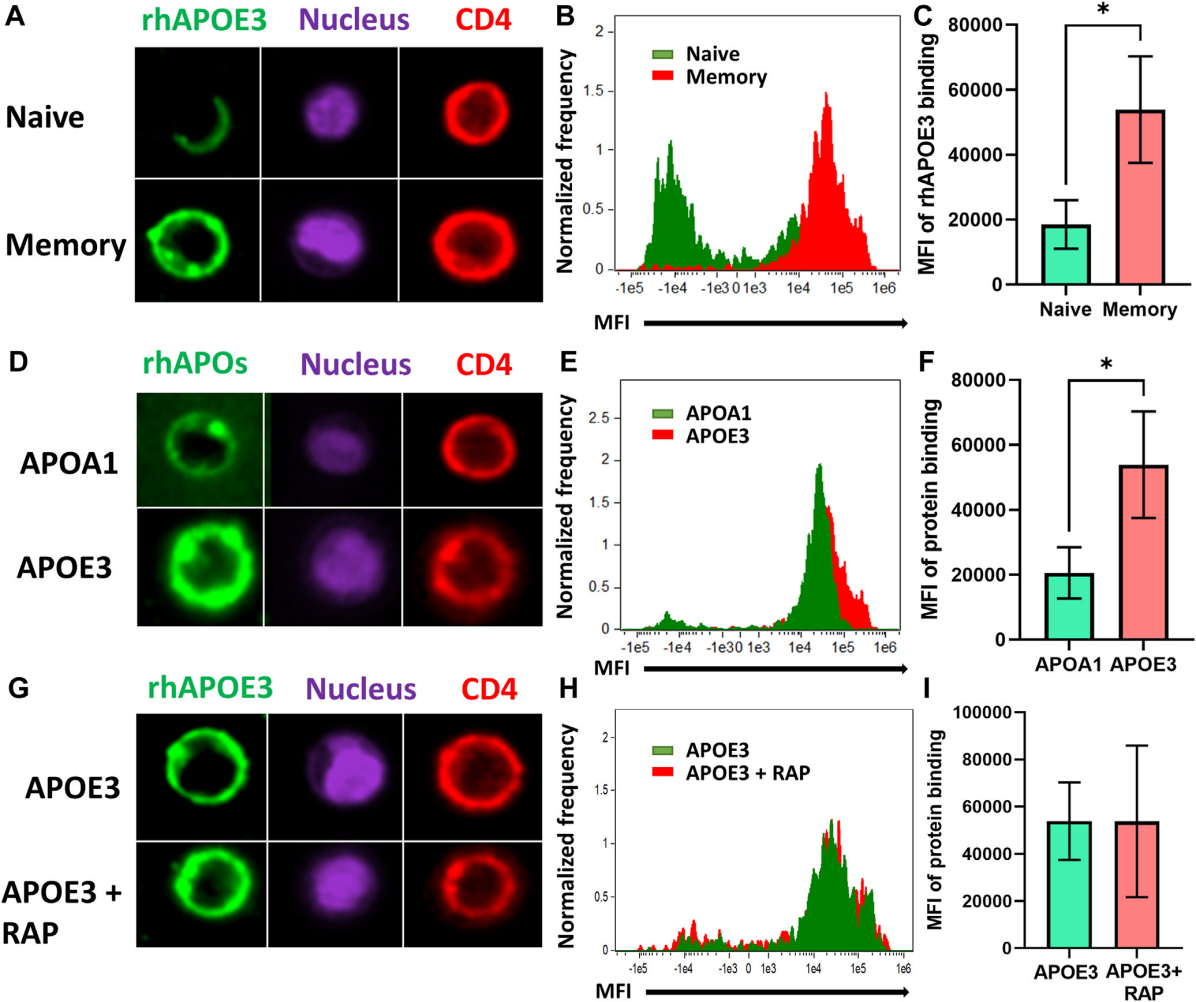
Total mTreg cells bind high amount of lipid-free rhAPOE3 but not rhAPOA1. Sorted nTreg (CD127−CD25+CD45RA+CD95−) and total mTreg (CD127−CD25+CD45RA−CD95+) were incubated with either rhAPOE3 or rhAPOA1 labeled with Alexa Fluor 488 for 1 h at 37°C in X-VIVO medium. Cells were stained with anti-CD4 (red) and NucBlue (purple) to visualize membrane and nucleus and analyzed by Imagestream. A: Representative example of each subset. B and C: MFI of fluorescent rhAPOE3 bound to either nTreg or mTreg. D: Representative example of one cell for each apolipoprotein. E and F: MFI of rhAPOE3- or rhAPOA1-bound mTreg. Asterisks indicate significant differences (∗*P* < 0.05) in *t*-test (n = 3). G: Representative example of one cell for rhAPOE3 or rhAPOE3 + RAP (LRPs inhibitor, 10 µM). H and I: MFI of rhAPOE3 bound to mTreg pretreated with or without RAP. Nonsignificant differences in *t*-test (n = 3).

Since HDL binding to mTreg is associated with its prosurvival effect (Fig. 2), we evaluated the ability of lipid-free rhAPOE3 to bind mTreg. We sorted nTreg and total memory, including the emTreg subset, from bulk Treg (see supplemental Fig. S1A for gating strategy) and exposed them to fluorescently labeled rhAPOE3 for 1 h. As a control, we used labeled rhAPOA1. Fig. 6A and supplemental Fig. S7A show that lipid-free rhAPOE3 bound to total mTreg but less so to nTreg. Total mTreg also displayed higher MFI of rhAPOE3 binding than nTreg (Fig. 6B,C). Furthermore, rhAPOE3 showed significantly higher binding to mTreg than the nonprotective rhAPOA1 (Fig. 6D–F and supplemental Fig. S7A). Next, we probed whether rhAPOE3-specific binding to mTreg was mediated by the APOE receptor, LRPs. mTreg subsets were treated with RAP, an inhibitor of LRPs (38), for 1 h before adding the fluorescent rhAPOE3. The treatment with RAP failed to affect rhAPOE3 binding to mTreg (Fig. 6G) and did not decrease MFI of rhAPOE3 binding to mTreg cell membrane (Fig. 6H,I). In addition, LRP inhibition by RAP did not significantly decrease HDL protective effect for mTreg and emTreg apoptosis (supplemental Fig. S7B), suggesting LRPs are not involved in HDL-APOE3 binding and signaling in Treg.

## Discussion

Plasma levels of HDL, at least as measured by their cholesterol content, are associated with protection from CVD, and it has become increasingly clear that immunological mechanisms may account for a substantial portion of the protection (39). We previously published that HDL had antiapoptotic effects in bulk Treg subset (6). Our results reported here clearly show that *i*) HDL primarily impacts the mTreg and emTreg subclasses, *ii*) it is the protein component of HDL, principally APOE, that mediates the lion’s share of this effect, and *iii*) theprotective effect of HDL is mediated, at least in part, via Akt-dependent signaling.

Treg homeostasis at steady state and during inflammation is controlled by the rate of proliferation versus apoptosis. The loss of Treg can directly cause autoimmunity (40) or accelerate the atherosclerotic process (7). Our new data using an ex vivo assay that reflects T-cell survival in vivo (29) show that Treg subsets are vastly different in terms of homeostasis, as nTreg subsets were not susceptible to apoptosis, whereas mTreg and emTreg subsets, which have a higher activation phenotype, go to apoptosis quickly. This is important as most of the literature on Treg survival has focused on the role of interleukin 2 (IL-2), although several studies suggest that blocking IL-2 minimally affects mTreg (41), suggesting other factors controlling Treg homeostasis besides IL-2 are important to understand. A better knowledge of the mechanisms involved in their survival, including those mediated by lipoproteins, could suggest new strategies to improve Treg control of inflammation.

HDL functionality is dependent on its apolipoprotein and lipid composition. Both apolipoprotein and lipid components appear to contribute to the HDL overall anti-inflammatory properties (3, 42). As opposed to the described antiapoptotic role of HDL lysosphingolipids for endothelial cells (42), we did not find any effect of HDL lipids in emTreg survival. Of note, a previous study implicated the sphingosine-1 phosphate receptor 1 (S1P_1_), the major HDL sphingolipid receptor, in Treg function in a mouse model (43). However, it is to be noted that, consistent with the role of S1P_1_ regulating T-cell exit from lymphoid tissues, this study demonstrated that S1P_1_ is critical for Treg localization to nonlymphoid tissues, but contrary to us, did not study circulating Treg homeostasis. In agreement with our new data, we previously found that blocking S1P_1_ did not have any effect on bulk Treg survival (6).

Many antiatherogenic functions of the HDL apolipoproteins have been attributed to APOA1. Therefore, it surprised us that APOA1 failed to mediate the Treg survival effect. De Souza et al. (44) found that HDL-APOA1 protected endothelial cells from apoptosis. Another study correlated APOA1 levels with Treg homeostasis, as Treg numbers from hypercholesterolemic mice (LDLr^−/−^, Apoa1^−/−^) were proportionally increased after the treatment with lipid-free APOA1 (16). However, this study did not demonstrate that APOA1 acted through a direct effect on Treg. Thus, HDL likely impact different cell types through different mechanisms (which may also include removal of cellular lipids), and these different activities synergize to mediate the full anti-inflammatory properties of HDL.

Our data identifying APOE as a major apolipoprotein involved in Treg antiapoptotic effect shed new mechanistic light on its known atheroprotective properties described in mice and human (45). Apoe^−/−^ mice are a well-established model for atherosclerosis; the lack of this apolipoprotein increases cholesterol levels, plaque formation, and sustained systemic inflammation (46). Consistent with our data, Treg also play an important protective role against the development of atherosclerosis in these mice, as their depletion exacerbates plaque formation and immune infiltration, whereas their adoptive transfer inhibits atherosclerosis (19, 20, 21). Moreover in humans, the homozygous APOE deficiency has been associated with severe atherosclerosis and premature CVD (47). There are three major human alleles of APOE (E2, E3, and E4), with their frequencies being 8.4, 77.9, and 13.7%, respectively, in the worldwide population (45, 48). These APOE genotypes are associated with different risks of atherosclerosis. APOE3 and APOE4 appear to have more beneficial effects and antiatherogenic properties compared with the rarest isoform APOE2. APOE2 is associated with increased levels of triglyceride-rich lipoproteins and type III hyperlipoproteinemia, which correlates with an early development of CVD (45). Importantly, we found that APOE3 and APOE4 are likely mediating the protective effect of HDL, whereas APOE2 was less protective. Similar to our findings, APOE3 was shown to protect endothelial cells from apoptosis by decreasing caspase-3/7 activation (49). APOE makes up ∼3% of total HDL protein and normal values of plasma concentration of APOE in normolipidemic individuals are between 20 and 70 μg/ml (37); it was striking that physiological concentrations of APOE3 and APOE4 containing HDL had the most significant effect in Treg survival. These data suggest that physiological concentrations of APOE3- and APOE4-containing HDL protect Treg from apoptosis, and this may be beneficial for CVD.

We previously proposed that HDLs were used as fuel to promote OXPHOS and fatty acid oxidation, after their uptake by Treg (6). Our new data suggest a different model, whereby HDL acts by signaling emTreg, in an Akt-dependent manner to control apoptosis as HDL apolipoproteins, and even lipid-free APOE3, can rescue emTreg from apoptosis, whereas the blockage of Akt attenuated the HDL prosurvival effect. In agreement with this new model, it has been described that HDL can inhibit apoptosis in endothelial cells by activating the Akt/endothelial nitric oxide synthase pathway (31, 50).

Here, we found that HDL mainly bound to mTreg, suggesting there may be a differential mechanism of HDL recognition between nTreg and mTreg. This may be due to a different expression of APOE receptors between the two Treg phenotypes. As lipid-free APOE cannot bind to LDLR (51, 52), Treg-HDL interactions are not likely to be LDLR mediated. APOE also interacts with other members of the LDL receptor family, the LRPs (51). However, the blockage of LRPs did not have any significant effect either on APOE3 binding or on HDL antiapoptotic capacity, suggesting that other receptors are involved in Treg-HDL interactions. Further studies will be needed to elucidate this mechanism.

The recent failure of HDL-cholesterol raising agents to provide clinical benefits for CVD has highlighted the need to better understand how they exert their pleiotropic effects on inflammation. Our study shows a new link between lipoprotein metabolism and Treg homeostasis that may be exploitable to reduce systemic inflammation during chronic metabolic diseases. It will be important to gain a more detailed mechanistic understanding of this effect with an eye toward developing APOE-containing synthetic lipoproteins for infusion or small molecules that mediate key parts of the pathway to enhance atheroprotection.

## Data availability

The data generated in the study are included in the article and supplemental data. Further inquiries can be directed to Claire.Chougnet@cchmc.org.

## Supplemental data

This article contains supplemental data.

## Conflict of interest

The authors declare that they have no conflicts of interest with the contents of this article.

## References

[1] Tang H., Xiang Z., Li L., Shao X., Zhou Q., You X. (2021). Potential role of anti-inflammatory HDL subclasses in metabolic unhealth/obesity. Artif. Cells Nanomed. Biotechnol..

[2] Stadler J.T., Lackner S., Mörkl S., Trakaki A., Scharnagl H., Borenich A. (2021). Obesity affects HDL metabolism, composition and subclass distribution. Biomedicines.

[3] Kontush A., Lindahl M., Lhomme M., Calabresi L., Chapman M.J., Davidson W.S. (2015). Structure of HDL: particle subclasses and molecular components. Handb. Exp. Pharmacol..

[4] Davidson W.S., Shah A.S., Sexmith H., Gordon S.M. (2022). The HDL proteome watch: compilation of studies leads to new insights on HDL function. Biochim. Biophys. Acta Mol. Cell Biol. Lipids.

[5] Trakaki A., Marsche G. (2021). Current understanding of the immunomodulatory activities of high-density lipoproteins. Biomedicines.

[6] Rueda C.M., Rodríguez-Perea A.L., Moreno-Fernandez M., Jackson C.M., Melchior J.T., Davidson W.S. (2017). High density lipoproteins selectively promote the survival of human regulatory T cells. J. Lipid Res..

[7] Ait-Oufella H., Salomon B.L., Potteaux S., Robertson A.K., Gourdy P., Zoll J. (2006). Natural regulatory T cells control the development of atherosclerosis in mice. Nat. Med..

[8] Baecher-Allan C., Wolf E., Hafler D.A. (2006). MHC class II expression identifies functionally distinct human regulatory T cells. J. Immunol..

[9] Dong S., Maiella S., Xhaard A., Pang Y., Wenandy L., Larghero J. (2013). Multiparameter single-cell profiling of human CD4+FOXP3+ regulatory T-cell populations in homeostatic conditions and during graft-versus-host disease. Blood.

[10] Miyara M., Yoshioka Y., Kitoh A., Shima T., Wing K., Niwa A. (2009). Functional delineation and differentiation dynamics of human CD4+ T cells expressing the FoxP3 transcription factor. Immunity.

[11] Sakaguchi S., Miyara M., Costantino C.M., Hafler D.A. (2010). FOXP3+ regulatory T cells in the human immune system. Nat. Rev. Immunol..

[12] Tamosiuniene R., Tian W., Dhillon G., Wang L., Sung Y.K., Gera L. (2011). Regulatory T cells limit vascular endothelial injury and prevent pulmonary hypertension. Circ. Res..

[13] Zhang Y., Gordon S.M., Xi H., Choi S., Paz M.A., Sun R. (2019). HDL subclass proteomic analysis and functional implication of protein dynamic change during HDL maturation. Redox Biol..

[14] Ruiz M., Okada H., Dahlbäck B. (2017). HDL-associated ApoM is anti-apoptotic by delivering sphingosine 1-phosphate to S1P1 & S1P3 receptors on vascular endothelium. Lipids Health Dis..

[15] Suc I., Escargueil-Blanc I., Troly M., Salvayre R., Nègre-Salvayre A. (1997). HDL and ApoA prevent cell death of endothelial cells induced by oxidized LDL. Arterioscler. Thromb. Vasc. Biol..

[16] Wilhelm A.J., Zabalawi M., Owen J.S., Shah D., Grayson J.M., Major A.S. (2010). Apolipoprotein A-I modulates regulatory T cells in autoimmune LDLr-/-, ApoA-I-/- mice. J. Biol. Chem..

[17] Gaddis D.E., Padgett L.E., Wu R., McSkimming C., Romines V., Taylor A.M. (2018). Apolipoprotein AI prevents regulatory to follicular helper T cell switching during atherosclerosis. Nat. Commun..

[18] Morton A.M., Koch M., Mendivil C.O., Furtado J.D., Tjønneland A., Overvad K. (2018). Apolipoproteins E and CIII interact to regulate HDL metabolism and coronary heart disease risk. JCI Insight.

[19] Mor A., Planer D., Luboshits G., Afek A., Metzger S., Chajek-Shaul T. (2007). Role of naturally occurring CD4+ CD25+ regulatory T cells in experimental atherosclerosis. Arterioscler. Thromb. Vasc. Biol..

[20] Mandatori S., Pacella I., Marzolla V., Mammi C., Starace D., Padula F. (2020). Altered Tregs differentiation and Impaired Autophagy correlate to atherosclerotic disease. Front. Immunol..

[21] Wang Z., Mao S., Zhan Z., Yu K., He C., Wang C. (2014). Effect of hyperlipidemia on Foxp3 expression in apolipoprotein E-knockout mice. J. Cardiovasc. Med. (Hagerstown).

[22] Xie J.J., Wang J., Tang T.T., Chen J., Gao X.L., Yuan J. (2010). The Th17/Treg functional imbalance during atherogenesis in ApoE(-/-) mice. Cytokine.

[23] Bligh E.G., Dyer W.J. (1959). A rapid method of total lipid extraction and purification. Can J. Biochem. Physiol..

[24] Bonomo E.A., Swaney J.B. (1988). A rapid method for the synthesis of protein-lipid complexes using adsorption chromatography. J. Lipid Res..

[25] Tubb M.R., Smith L.E., Davidson W.S. (2009). Purification of recombinant apolipoproteins A-I and A-IV and efficient affinity tag cleavage by tobacco etch virus protease. J. Lipid Res..

[26] Cooke A.L., Morris J., Melchior J.T., Street S.E., Jerome W.G., Huang R. (2018). A thumbwheel mechanism for APOA1 activation of LCAT activity in HDL. J. Lipid Res..

[27] Scanu A.M., Edelstein C. (1971). Solubility in aqueous solutions of ethanol of the small molecular weight peptides of the serum very low density and high density lipoproteins: relevance to the recovery problem during delipidation of serum lipoproteins. Anal. Biochem..

[28] Maiorano J.N., Davidson W.S. (2000). The orientation of helix 4 in apolipoprotein A-I-containing reconstituted high density lipoproteins. J. Biol. Chem..

[29] Hildeman D.A., Zhu Y., Mitchell T.C., Bouillet P., Strasser A., Kappler J. (2002). Activated T cell death in vivo mediated by proapoptotic bcl-2 family member bim. Immunity.

[30] Benbrook D.M., Masamha C.P. (2011). The pro-survival function of Akt kinase can be overridden or altered to contribute to induction of apoptosis. Curr. Cancer Drug Targets.

[31] Nofer J.R., Levkau B., Wolinska I., Junker R., Fobker M., von Eckardstein A. (2001). Suppression of endothelial cell apoptosis by high density lipoproteins (HDL) and HDL-associated lysosphingolipids. J. Biol. Chem..

[32] Xing Y., Lin N.U., Maurer M.A., Chen H., Mahvash A., Sahin A. (2019). Phase II trial of AKT inhibitor MK-2206 in patients with advanced breast cancer who have tumors with PIK3CA or AKT mutations, and/or PTEN loss/PTEN mutation. Breast Cancer Res..

[33] Rosales C., Davidson W.S., Gillard B.K., Gotto A.M., Pownall H.J. (2016). Speciated high-density lipoprotein biogenesis and functionality. Curr. Atheroscler. Rep..

[34] Melchior J.T., Street S.E., Vaisar T., Hart R., Jerome J., Kuklenyik Z. (2021). Apolipoprotein A-I modulates HDL particle size in the absence of apolipoprotein A-II. J. Lipid Res..

[35] Fan Q., Liu Y., Rao J., Zhang Z., Xiao W., Zhu T. (2019). Anti-atherosclerosis effect of Angong Niuhuang Pill via regulating Th17/Treg immune balance and inhibiting chronic inflammatory on ApoE(-/-) mice model of early and Mid-Term atherosclerosis. Front. Pharmacol..

[36] Sharma M., Schlegel M.P., Afonso M.S., Brown E.J., Rahman K., Weinstock A. (2020). Regulatory T cells license macrophage pro-resolving functions during atherosclerosis regression. Circ. Res..

[37] Kaneva A.M., Bojko E.R., Potolitsyna N.N., Odland J.O. (2013). Plasma levels of apolipoprotein-E in residents of the European North of Russia. Lipids Health Dis..

[38] LaDu M.J., Shah J.A., Reardon C.A., Getz G.S., Bu G., Hu J. (2001). Apolipoprotein E and apolipoprotein E receptors modulate A beta-induced glial neuroinflammatory responses. Neurochem. Int..

[39] Norata G.D., Pirillo A., Ammirati E., Catapano A.L. (2012). Emerging role of high density lipoproteins as a player in the immune system. Atherosclerosis.

[40] Pierson W., Cauwe B., Policheni A., Schlenner S.M., Franckaert D., Berges J. (2013). Antiapoptotic Mcl-1 is critical for the survival and niche-filling capacity of Foxp3⁺ regulatory T cells. Nat. Immunol..

[41] Buszko M., Shevach E.M. (2020). Control of regulatory T cell homeostasis. Curr. Opin. Immunol..

[42] Norata G.D., Catapano A.L. (2005). Molecular mechanisms responsible for the antiinflammatory and protective effect of HDL on the endothelium. Vasc. Health Risk Manag..

[43] Eken A., Duhen R., Singh A.K., Fry M., Buckner J.H., Kita M. (2017). S1P(1) deletion differentially affects TH17 and Regulatory T cells. Sci. Rep..

[44] de Souza J.A., Vindis C., Nègre-Salvayre A., Rye K.A., Couturier M., Therond P. (2010). Small, dense HDL 3 particles attenuate apoptosis in endothelial cells: pivotal role of apolipoprotein A-I. J. Cell. Mol. Med..

[45] Valanti E.K., Dalakoura-Karagkouni K., Sanoudou D. (2018). Current and emerging reconstituted HDL-apoA-I and HDL-apoE approaches to treat atherosclerosis. J. Pers. Med..

[46] Lo Sasso G., Schlage W.K., Boué S., Veljkovic E., Peitsch M.C., Hoeng J. (2016). The Apoe(-/-) mouse model: a suitable model to study cardiovascular and respiratory diseases in the context of cigarette smoke exposure and harm reduction. J. Transl. Med..

[47] Schaefer E.J., Gregg R.E., Ghiselli G., Forte T.M., Ordovas J.M., Zech L.A. (1986). Familial apolipoprotein E deficiency. J. Clin. Invest..

[48] Liu C.C., Kanekiyo T., Xu H., Bu G. (2013). Apolipoprotein E and Alzheimer disease: risk, mechanisms and therapy. Nat. Rev. Neurol..

[49] DeKroon R.M., Mihovilovic M., Goodger Z.V., Robinette J.B., Sullivan P.M., Saunders A.M. (2003). ApoE genotype-specific inhibition of apoptosis. J. Lipid Res..

[50] Mineo C., Yuhanna I.S., Quon M.J., Shaul P.W. (2003). High density lipoprotein-induced endothelial nitric-oxide synthase activation is mediated by Akt and MAP kinases. J. Biol. Chem..

[51] Ruiz J., Kouiavskaia D., Migliorini M., Robinson S., Saenko E.L., Gorlatova N. (2005). The apoE isoform binding properties of the VLDL receptor reveal marked differences from LRP and the LDL receptor. J. Lipid Res..

[52] Zhao N., Liu C.C., Qiao W., Bu G., Apolipoprotein E. (2018). Receptors, and modulation of Alzheimer's disease. Biol. Psychiatry.

