# Supplementary material for: Apolipoprotein E-containing HDL decreases caspase-dependent apoptosis of memory regulatory T lymphocytes
Source: J Lipid Res. 2023 Aug 12;64(9):100425. doi: 10.1016/j.jlr.2023.100425 (PMC10507648; doi:10.1016/j.jlr.2023.100425)
Supplement: Supplemental Figures S1–S7 [file mmc1.pdf]

**Apolipoprotein E-containing HDL decrease caspase-dependent apoptosis of memory regulatory T lymphocytes.**

Laura Atehortua[1], Scott E Street[2], Jamie Morris[2], Nicholas Bedel[2], W. Sean Davidson[2] and Claire A. Chougnet[1]

1. Division of Immunobiology, Cincinnati Children's Hospital Research Foundation, University of Cincinnati College of Medicine, Cincinnati, OH.

2. Division of Experimental Pathology, Department of Pathology and Laboratory Medicine, University of Cincinnati, Cincinnati, OH

## Supplementary figures.

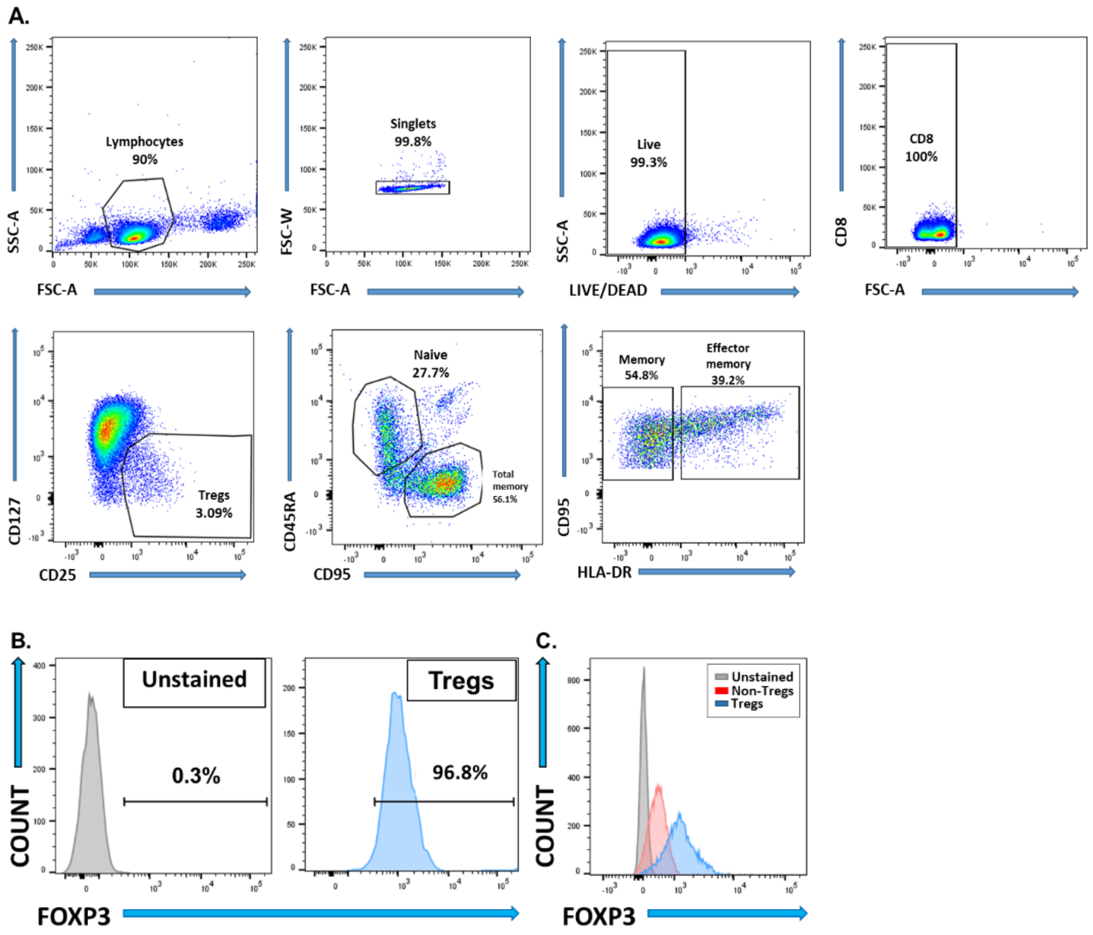

**Figure S1. Fluorescence cell sorting gating strategy**. **A.** CD4+ T cells were purified by negative bead selection from PBMCs and stained with fluorescently labeled Ab. Treg were identified as CD8-CD127-CD25+ within the singlets, live/dead negative cells. From bulk Treg, Treg subsets were identified as naïve Treg CD45RA+CD95-HLA-DR-, memory Treg CD45RA-CD95+HLA-DR-, and effector memory CD45RA-CD95+HLA-DR+. A representative example of >5 experiments is shown. Representative FOXP3 staining of **B.** Unstained cells vs Tregs and **C.** FOXP3 post-sorting analysis Treg vs non-Treg subsets

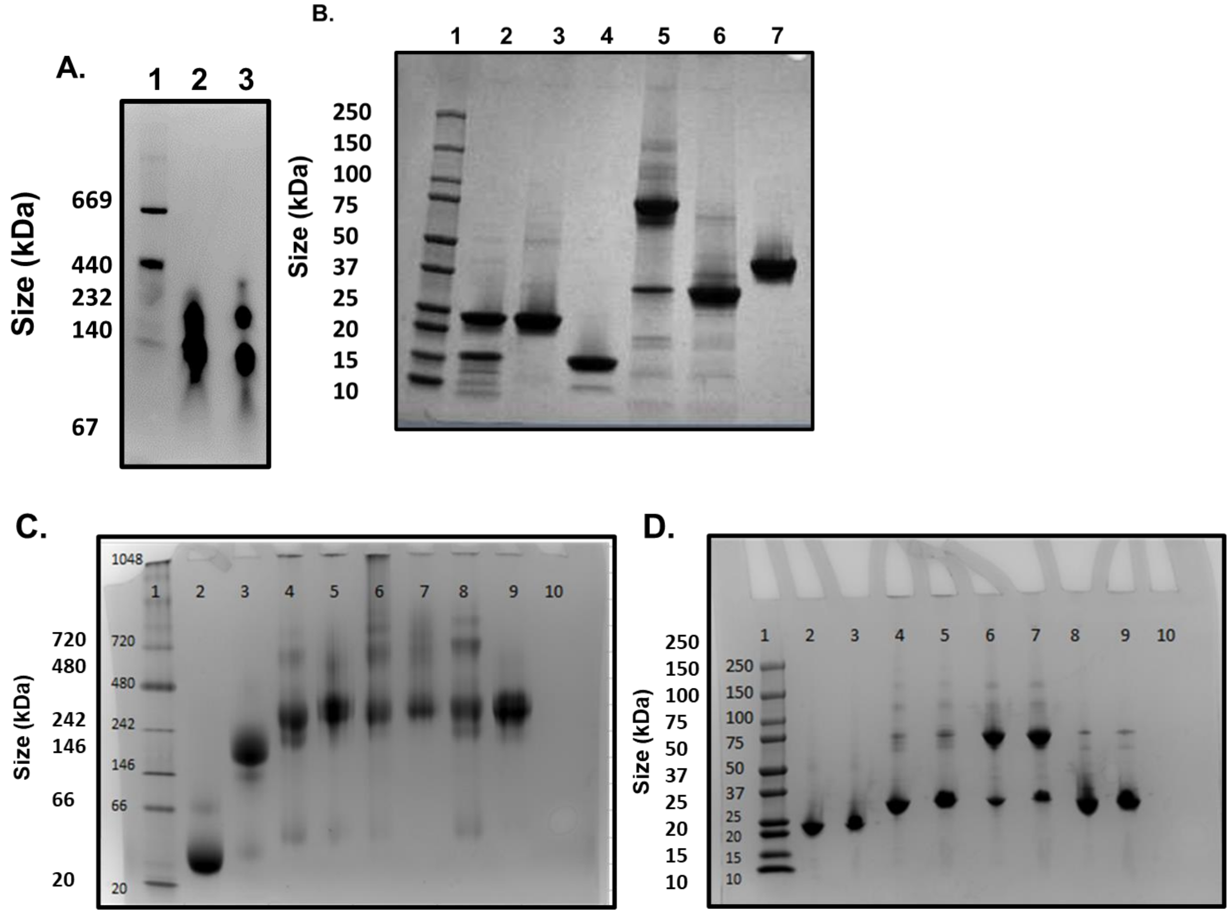


**Figure S2. Reconstitution of HDL lipids and apolipoproteins into rHDL particles. A.** Native NDGGE analysis of:Lane 1: MW standards, lane 2: 85:1 POPC: APOA1, lane 3: 72 : 13 : 1 POPC : HDL lipids : APOA1. **B.** SDS-PAGGE analysis of: Lane 1: MW standards, lane 2: apo HDL disc, lane 3: rAPOA1 disc, lane 4: pAPOA2 disc, lane 5: rHDL-APOE3 disc, lane 6: rHDL-APOE4 disc and lane 7: rHDL-APOA4 disc. This gel was run under non-reducing conditions. APOE3 tends to form disulfide dimers whereas

APOE4 tends to remain as monomers post reconstitution into HDL, hence the difference in size of the two bands on the gel. **C.** Native PAGE and **D.** SDS 4-15% of lane 1: ladder, lane 2: lipid free rhAPOA1, lane 3: rHDL-APOA1 disc, lane 4: lipid free rhAPOE2, lane 5: rHDL-APOE2 disc, lane 6: lipid free rhAPOE3, lane 7: rHDL-APOE3 disc, lane 8: lipid free rhAPOE4 and lane 9: rHDL-APOE4 disc.

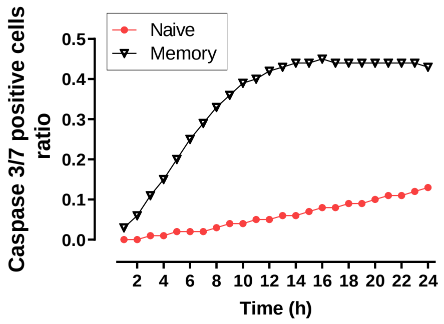


**Figure S3. Memory Treg express higher caspase 3/7 levels after 14h of incubation than naïve Treg.** Bulk Treg were cultured in X-VIVO in presence of caspase 3/7 green reagent and followed for 24 h by time-lapse microscopy. Based on the expression of CD45RO, Naïve (CD45RO-) were separated out from memory (CD45RO+) Treg.

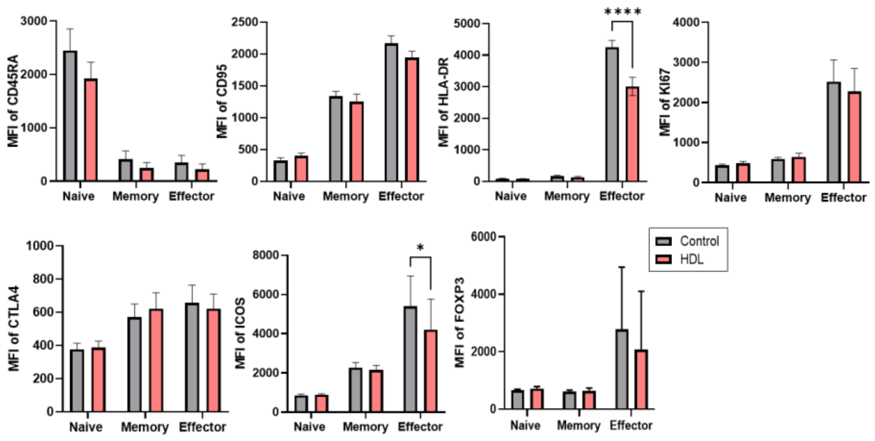


**Figure S4. Treg subsets keep their phenotype after HDL treatment**. Purified bulk Treg were incubated overnight with or without pooled HDL (700 ug/ml of protein) in X-VIVO medium. Treg were stained with antibodies against: CD45RA, CD95, HLA-DR, KI67, CTLA4, ICOS and FOXP3, and analyzed by flow

cytometry. Graphs show the MFI of each marker, asterisks indicate significant differences in Two-way ANOVA, (*p<0.05, ***p-value< 0.0005) (n= 6).

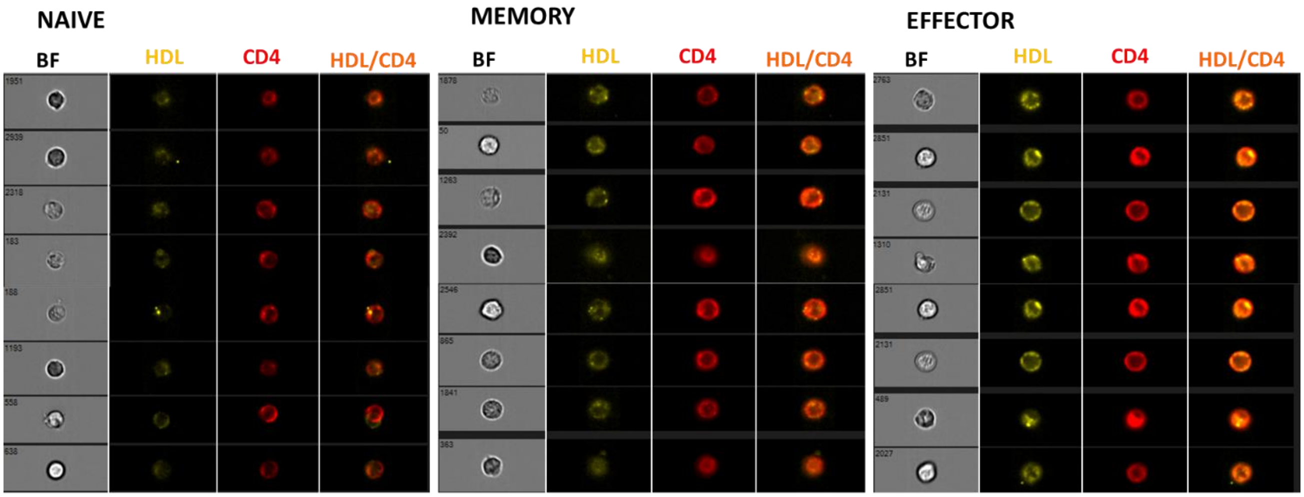


**Figure S5. HDL preferentially binds to memory and effector memory Treg.** Purified Treg subsets were cultured with HDL-Dil for 1h at 37°C in X-VIVO medium. Cells were stained with anti-CD4 (red) to visualize membrane and analyzed by Imagestream Representative random images of naïve, memory and effector memory Treg.

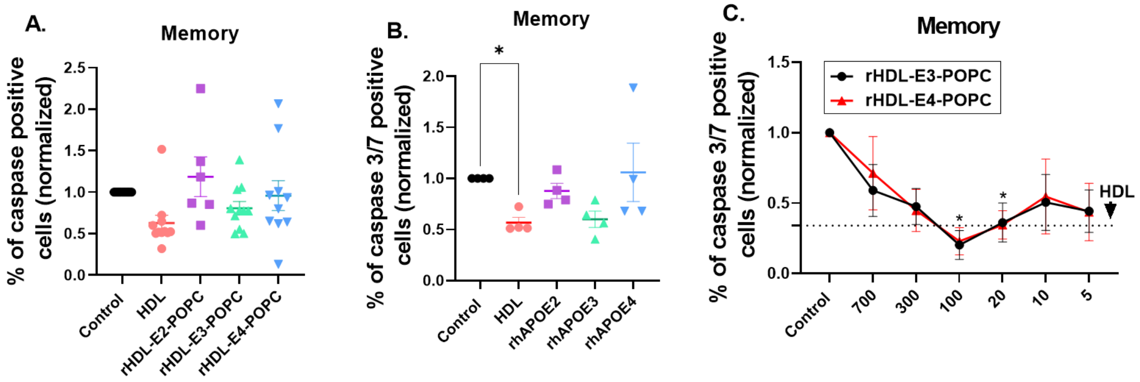


**Figure S6. rHDL-APOE dics decrease apoptosis in memory Treg.** Purified bulk Treg were incubated with total HDL or rAPOE (2, 3, 4) discs overnight in X-VIVO medium. **A-C.** Caspase 3/7 expression of Memory Treg was analyzed by flow cytometry and the data was normalized against the control (PBS). **B.**

Asterisks indicate significant differences in One-way ANOVA, (*p<0.05) (n=4). **C.** Dotted line shows the mean of total HDL treatment (700 ug/ml of protein). Asterisks indicate significant differences in Two-way ANOVA (control vs rHDL-E-POPC) (*p<0.05) (n=5).

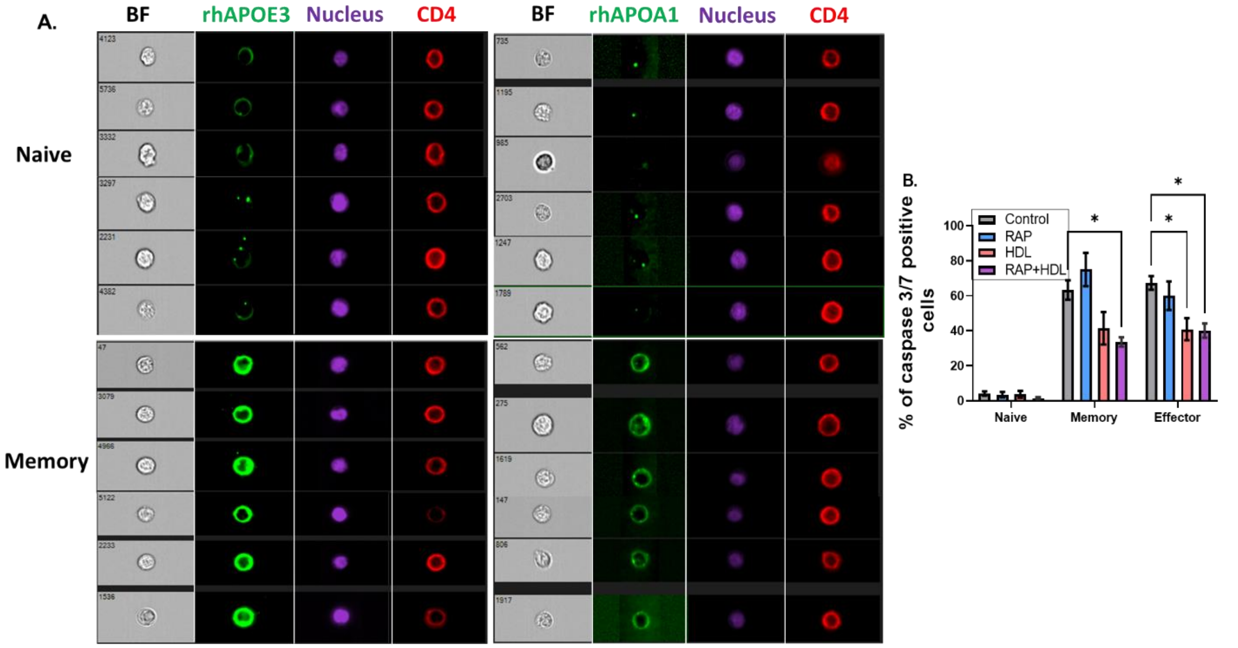


**Figure S7. Lipid free rhAPOE3 preferentially binds to memory Treg, but not through a LRPs-dependent mechanism. A.** Purified naïve and memory Treg were cultured with rhAPOE3 or rhAPOA1 labeled with Alexa fluor 488 for 1h at 37°C in X-VIVO medium. Cells were stained with anti-CD4 (red) to visualize membrane and nuc blue to visualize the nucleus and analyzed by Imagestream. Representative random images of naïve and memory Treg. **B.** Purified bulk Treg were treated with RAP (10 µM) for 1 h before adding the HDL (700 µg/ml of protein) overnight in X-VIVO medium. Caspase-3/7 expression was measured by flow cytometry in nTreg, mTreg and emTreg. Asterisks indicate significant differences (*p<0.05) two-way ANOVA, n= 4.
